# Yellow Fever Autopsies

**Published:** 1848-11

**Authors:** B. Rush Mitchell

**Affiliations:** U. S. Navy


### MEDICAL EXAMINER,

AND

### RECORD OF MEDICAL SCIENCE.

NEW SERIES.—NO. XLVII.—NOVEMBER, 1848.

### ORIGINAL COMMUNICATIONS.

*Yellow Fever Autopsies.* By B. RUSH MITCHELL, M. D., U. S. Navy.

If facts be important in medicine, then the contribution of them, even if the only effect of the communication be to establish more firmly long cherished views, cannot be esteemed altogether unprofitable. Influenced by considerations like these, we present to the profession the results of two post-mortem examinations made of yellow fever patients at the Naval Hospital, Salmadina Islands, near Vera Cruz, during the summer of 1847. Sickness, in part, prevented the writer from making more; although the greatest impediment was the paucity of dead cases.

*Case 1st.* J. R. Maine. Death occurring after six days illness. Autopsy ten hours after death.

*Results.*—Body full and plump, very rigid; cheeks of a bronzed appearance; about the ears and posterior to them, skin of a dark purple.

Abdomen contains a large quantity of effused serum. The stomach and bowels much inflated, the peritoneum presents marks of old adhesions, and is of a purplish colour externally. The stomach contains eight ounces of semi-consistent fluid, of a coffeeground hue and appearance; its mucous membrane is slightly injected throughout; and in the lesser curvature there is evident softening. The duodenum presents the same appearances as the stomach. The gall bladder is of natural size, and contains one ounce of very viscid black bile. The liver is natural in size, softened in texture, and of a mahogany colour. The spleen is of natural size, but much softened. The bladder is highly inflamed, and much distended with reddish-brown urine.

The lungs present nothing abnormal. The pericardium is full of serum. The heart is enlarged, thickened and fatty. There are old adhesions of the pleura.

Brain.—A large quantity of serum, evident upon removing the top of the cranium. The meninges are much injected and adherent. The substance of the cerebrum is more solid than in health, while the cerebellum is softened and very much injected.

This patient was *delirious* for two days prior to death; and was a fair illustration of those cases of yellow fever where the head lesions are those of most fatal import.

*Case 2d.*—W. Savage, seaman, died after eight days illness; three days sick before entering hospital. Autopsy 12 hours after death.

*Appearances.*—Body. No emaciation of any consequence. Eyes and skin of a saffron hue.

Pleura pale, and some traces of old adhesions. Static congestion of both lungs. Heart and pericardium perfectly normal.

Liver of usual density of structure, but one-third larger than usual, of a dark olive colour, with white streaks intermingled.

Gall bladder full of inky and ropy matter. Stomach more than half distended, and nearly full of semi-fluid and black matter. Its mucous coat is brilliantly red, when the black adherent matter is removed; and about the lesser curvature there is softening. To these appearances must be added great contraction and hardening of the pylorus.

The duodenum is much inflamed, thickened, and contracted in calibre; in a few places there is slight ulceration, chiefly in the upper third. The spleen is about twice the normal size, and of the consistence of clotted blood. The remaining bowels are much congested, and more or less filled with brownish fluid matter. The bladder is normal, and contains about half oz. of pale urine. The right kidney is much inflamed internally; and there are patches of lymph on the periphery of both.

The cerebrum and cerebellum are much softened; and are of the consistence of fresh made cheese. No serum in the ventricles. The membranes are pale and dry.

This case I esteem one of the class in which the abdominal lesions are most to be dreaded. At no period of his illness was this patient delirious; and although the autopsy revealed softening of the brain, yet there was no cerebral symptom present except coma, which came on an hour before death. In truth, I see no reason for believing that the lesions of the brain, noted in the last patient, had any necessary connexion with the disease which caused his death; and I am inclined to believe the softening of the brain to have been the gradual and progressive result of causes other than yellow fever.

The two post-mortems, it will be observed, differ much in the brainial lesions which they exhibit; as in truth they do in other points. Yet there are some important things common to the two; and as both were undoubted cases of yellow fever, it may be well to sum up and examine the points of agreement. The most prominent of these points of agreement is the condition of the stomach. In both it was evidently inflamed and softened, and this most marked about the lesser curvature. In both, this organ contains a dark-brown or black semi-fluid. The duodenum, too, is in both cases the subject of inflammation. The gall-bladder in both is full of inky, ropy bile. The liver in both is the subject of lesion, either in regard to colour or texture. The spleen is in both cases softened. The urinary apparatus is in both cases the subject of lesion; in the one there is inflammation of the bladder; in the other, of the kidneys.

If we leave the abdomen, the parallelism of the two post-mortems ceases, although we find no difference as respects the lungs and pleura. The differences are in the heart and the brain. The first named we are disposed to look upon as entirely disconnected from the disease; for the period of illness was too short for fatty enlargement of the heart; and the circumstances were not favorable to its depositions. And while we are indisposed to consider the softening of the brain in the second case as the product of the yellow fever, we cannot esteem the marked traces of inflammation in the first in a similar light. On the contrary, we view them as a legitimate result of yellow fever in a constitution in which the nervous temperament predominates. In all the cases that we have seen, we have invariably found strongly marked head symptoms in all cases of yellow fever attacking persons in whom, from constitutional peculiarity or from extrinsic causes, the brain was very active. So true is this, that it is practicable to tell, *a priori*, from the habits of the patient, whether the head or the abdominal symptoms will most predominate in his case. Thus, at Salmadina hospital, while the officers died with the head symptoms the most prominent, the reverse was generally true of the men.

Still, in all cases where the head symptoms predominated, they did not appear simultaneously with the access of the fever; but became manifested during its course, and usually about two days before death. They appeared to us to be the result of transferred irritation from the abdomen to the head. And the reason why this transferrence takes place so readily, we imagine to be the fact, that the cause of yellow fever first affects the nervous system, the spinal in particular, as evidenced by the intense pain in the back, present at the beginning of *all* cases.

We believe, and the results of these post-mortems indicate it, that the chief seat of yellow fever, the grand object of its assault, is, the various parts within the abdomen; and if we succeed in defeating the disease in this locality, there is no fear of a transferrence of disease to the head, or of a termination of the case in death. How these desirable ends may be attained, is a most important query in practical medicine. To solve it perfectly and satisfactorily, there is required an *honest, truthful, capable and perfectly unbiassed* observer. When will such an one be found?

